# Intracellular Secretory Leukoprotease Inhibitor Modulates Inositol 1,4,5-Triphosphate Generation and Exerts an Anti-Inflammatory Effect on Neutrophils of Individuals with Cystic Fibrosis and Chronic Obstructive Pulmonary Disease

**DOI:** 10.1155/2013/560141

**Published:** 2013-08-29

**Authors:** Emer P. Reeves, Nessa Banville, Dorothy M. Ryan, Niamh O'Reilly, David A. Bergin, Kerstin Pohl, Kevin Molloy, Oliver J. McElvaney, Khalifah Alsaleh, Ahmed Aljorfi, Osama Kandalaft, Eimear O'Flynn, Patrick Geraghty, Shane J. O'Neill, Noel G. McElvaney

**Affiliations:** Respiratory Research Division, Department of Medicine, Royal College of Surgeons in Ireland, Education & Research Centre, Beaumont Hospital, Dublin 9, Ireland

## Abstract

Secretory leukoprotease inhibitor (SLPI) is an anti-inflammatory protein present in respiratory secretions. Whilst epithelial cell SLPI is extensively studied, neutrophil associated SLPI is poorly characterised. Neutrophil function including chemotaxis and degranulation of proteolytic enzymes involves changes in cytosolic calcium (Ca^2+^) levels which is mediated by production of inositol 1,4,5-triphosphate (IP_3_) in response to G-protein-coupled receptor (GPCR) stimuli. The aim of this study was to investigate the intracellular function of SLPI and the mechanism-based modulation of neutrophil function by this antiprotease. Neutrophils were isolated from healthy controls (*n* = 10), individuals with cystic fibrosis (CF) (*n* = 5) or chronic obstructive pulmonary disease (COPD) (*n* = 5). Recombinant human SLPI significantly inhibited fMet-Leu-Phe (fMLP) and interleukin(IL)-8 induced neutrophil chemotaxis (*P* < 0.05) and decreased degranulation of matrix metalloprotease-9 (MMP-9), hCAP-18, and myeloperoxidase (MPO) (*P* < 0.05). The mechanism of inhibition involved modulation of cytosolic IP_3_ production and downstream Ca^2+^ flux. The described attenuation of Ca^2+^ flux was overcome by inclusion of exogenous IP_3_ in electropermeabilized cells. Inhibition of IP_3_ generation and Ca^2+^ flux by SLPI may represent a novel anti-inflammatory mechanism, thus strengthening the attractiveness of SLPI as a potential therapeutic molecule in inflammatory airway disease associated with excessive neutrophil influx including CF, non-CF bronchiectasis, and COPD.

## 1. Introduction

The ability of neutrophils to mobilise rapidly to a site of infection is paramount to successful elimination of foreign microbes; however, an excessive infiltrative response can elicit extensive tissue damage. Indeed, neutrophil migration to the airways is a feature of a number of chronic pulmonary disorders including cystic fibrosis (CF) and chronic obstructive pulmonary disease (COPD), where neutrophil elastase (NE) is largely responsible for protease-mediated damage in the lung. A gradient of attractant molecules, including the bacterial chemotactic peptide fMLP signalling through FPR1 and tissue derived IL-8 signalling via the chemokine receptors CXCR1 and CXCR2, attract neutrophils to migrate towards the site of tissue infection in a directional manner referred to as chemotaxis. Upon cell migration, the normally spherical cell undergoes lateral polarization to form a leading edge and a trailing tail [1]. To achieve this morphological change many processes are initiated including cytoskeletal reorganisation with protein polymers of filamentous actin, microtubules, and intermediate filaments forming at the leading edge and dissolution of polymers occurring at the rear [2]. 

Molecular regulation of cytoskeletal rearrangements during neutrophil signalling associated with chemotaxis and degranulation of antimicrobial enzymes, requires an increase in cytosolic Ca^2+^ levels [3]. This increase is due to the release of Ca^2+^ from the cell's internal store (the calciosome) or influx of Ca^2+^ from the extracellular environment. Signalling via fMLP or IL-8 GPCRs results in activation of the *β* isoform of phospholipase C, in turn yielding production of inositol 1,4,5 triphosphate (IP_3_) [4]. IP_3_ occupancy of receptors on calciosomes induces a rapid release of stored Ca^2+^, and in a variety of cell types this cascade has been proposed as a potential target for the treatment of a number of diseases including heart failure and arrhythmias [5, 6].

Serine protease inhibitors such as SLPI act locally to maintain a protease/antiprotease balance thereby preventing protease mediated tissue destruction [7]. Disruption of this balance is responsible for much of the extracellular matrix and subsequent lung tissue damage evident in neutrophil driven lung diseases [8, 9]. SLPI is a well-characterised member of the trappin gene family of proteins [10]. It is a highly basic cationic protein that is produced by epithelial cells of the respiratory tract and is also produced by phagocytic neutrophils [11]. SLPI inhibits a broad range of serine proteases including NE, cathepsin G, trypsin, and tryptase. Moreover, SLPI possesses anti-inflammatory characteristics and in monocytes has been shown to inhibit *lipopolysaccharide* (LPS)- and lipoteichoic acid-induced nuclear factor (NF)-*κ*B activation and can compete with p65 for binding to NF-*κ*B binding sites [12, 13]. Moreover, SLPI has been shown to directly bind to bacterial LPS thereby down-regulating production of proinflammatory mediators [14, 15]. However, an area that has received less attention is the role of neutrophil-derived SLPI in modulating inflammatory responses. 

Studies have localised SLPI within the cytosol [11] and also in secondary granules of neutrophils, in turn coreleased with lactoferrin [16]. Moreover, the release of SLPI from neutrophils upon phorbol ester (PMA) activation suggests that neutrophil-derived SLPI may regulate the protease antiprotease balance at sites of tissue inflammation [11]. However, the aim of this study was to investigate the intracellular function of SLPI within resting and activated neutrophils. This study has revealed a novel anti-inflammatory role for this antiprotease and demonstrated the ability of SLPI to modulate neutrophil migration and degranulation by inhibiting IP_3_ production and Ca^2+^ ion mobilisation. 

## 2. Methods

### 2.1. Chemicals and Reagents

All chemicals and reagents including inositol 1,4,5-triphosphate were purchased from Sigma-Aldrich, St. Louis, MO, USA, unless indicated otherwise. The concentration of SLPI in neutrophil cytosol was measured using the Quantikine Human SLPI Immunoassay (R & D Systems, Abingdon, Oxon, UK). Recombinant human SLPI (rhSLPI) was obtained from R & D Systems, and recombinant calpain was obtained from Calbiochem (La Jolla, CA, USA). Human NE was purchased from Elastin Products Company, Inc. (Owensville, MO, USA). The IP-One HTRF assay kit was purchased from Cisbio Bioassays (Bedford, MA 01730, USA).

### 2.2. Study Groups

Control volunteers (*n* = 10, mean age 35.1 ± 1.8) had no underlying medical illnesses and were not receiving any medication. Prior to recruitment patients with CF (*n* = 5) were exacerbation-free over the preceding 6-week period, and patients with COPD were being treated for an acute exacerbation (*n* = 5). There were 2/5 and 3/5 males recruited to the CF and COPD study, respectively. The mean age was 22.5 ± 2.7 and 56.5 ± 2.7 years for the CF and COPD groups, respectively. Informed patient consent was obtained for all procedures, and ethical approval for the use of blood samples from CF and COPD individuals was obtained from the Beaumont Hospital Ethics Review Board.

### 2.3. Preparation of Human Neutrophils and Cell Fractionation

Blood was obtained from all donors in 7.5 mL heparinised S-monovette tubes (Sarstedt Ltd, Ireland), and neutrophils were purified by dextran sedimentation and Lymphoprep (Axis-Shield PoC AS, Norway) centrifugation as previously described [17]. Purified cells were resuspended in phosphate-buffered saline (PBS) (pH 7.4) containing 5 mM glucose (PBSG) and used immediately. The purity of the neutrophil population was confirmed by flow cytometry measuring the neutrophil membrane marker CD16b and was found to be >99% [18]. All *in vitro* experiments were performed at 37°C. Cells were either left unstimulated (control) or activated with PMA (1.6 *μ*M), fMLP (1 *μ*M), and/or IL-8 (1.2 nM, R & D Systems) for the indicated time points. 

For cell fractionation studies, isolated neutrophils were suspended in Lamberts Break Buffer (LBB) (10 mM KCl, 3 mM NaCl, 4 mM MgCl_2_, and 10 mM piperazine-N,N′-bis(2-ethanesulfonic acid) (PIPES); pH 7.2) containing 10% (w/w) sucrose and the following protease inhibitors: 13 *μ*M aprotinin, 5 mM benzamidine, 0.15 mM N*α*-Tosyl-L-lysine chloromethyl ketone hydrochloride (TLCK), 0.5 mM N-p-Tosyl-L-phenylalanine chloromethyl ketone (TPCK), 20 mM N-(Methoxysuccinyl)-Ala-Ala-Pro-Val-chloromethyl ketone (MeOSuc-AAPV-CMK), 10 *μ*M soybean trypsin inhibitor (SBTI), orthophenanthroline, and 0.2 M pefabloc. Cells were sonicated 3 times for 5 sec on ice and centrifuged at 500 ×g at 4°C for 5 min to generate a post nuclear supernatant (PNS). The PNS was overlaid on discontinuous sucrose gradients of 17.5 and 35% (w/w) dissolved in LBB. This was centrifuged at 137,000 ×g for 45 min at 4°C. The cytosolic fraction was removed from above the 17% (w/w) sucrose layer, and membrane fraction was recovered from the top of the 34% (w/w) sucrose layer. The pellet (containing primary and secondary granules) was resuspended in 10% (w/w) sucrose and overlaid on a discontinuous sucrose gradient of 30, 43, and 55% (w/w). This was centrifuged at 137,000 ×g for 1 h at 4°C. Secondary and primary granules were harvested from above the 43% (w/w) and 55% (w/w) sucrose layers, respectively, as previously described [18]. The concentration of protein present in each purified fraction was quantified by use of a BCA Protein Assay Kit (Thermo Scientific) according to the manufacturer's instructions.

Quantification of F- and G-actin was carried out as previously described [19]. In brief, neutrophils were either untreated or treated with SLPI (480 nM) or wortmannin (100 nM) for 10 min and then left unactivated or activated with IL-8 (1.2 nM) and fMLP (1 *μ*M) for 10 min. Neutrophils were lysed within an F-actin stabilisation buffer containing ATP (1 mM) and protease inhibitors (as indicated above) for 10 min at 37°C. Clarified cell lysates were centrifuged for 2 h at 100,000 ×g. The supernatant was removed and the pellet was resuspended in an equal volume of buffer containing cytochalasin D (10 mM) and incubated on ice for 1 h. An equal volume of supernatant and pellet was electrophoresed and Western blotted for actin employing a monoclonal anti-actin antibody (1.0 *μ*g/mL; Millipore, Billerica, MA, USA).

### 2.4. Neutrophil Uptake Assays

Native neutrophil cytosolic SLPI levels were determined by ELISA (SLPI Human ELISA, Cambridge Bioscience, UK). Uptake of exogenous recombinant human SLPI (rhSLPI; 480 nM) by cells (2 × 10^5^/mL in PBSG) was performed at 37°C for 10 min. After incubation, the SLPI loaded cells were washed with ice cold PBSG and neutrophil levels of cytosolic SLPI measured by ELISA. To determine the mechanism of SLPI uptake, cell (2 × 10^5^/mL in PBSG) incubations were performed at 4°C for 10 min or in the presence of the endocytosis inhibitors sodium azide (NaN_3_; 15 mM) and sodium fluoride (NaF; 10 mM) as previously described [20].

### 2.5. SDS-PAGE and Western Blot Analyses

Samples were subjected to SDS-PAGE under denaturing conditions on 4–12% (w/v) NuPAGE gels (Invitrogen, Carlsbad, CA, USA) following the manufacturer's instructions. Routinely 25 *μ*g of neutrophil cytosolic protein, 10 *μ*g of membrane protein, 5 *μ*g secondary granule protein, and 10 *μ*g of primary granule protein was loaded on each gel. After electrophoresis gels were stained by Coomassie blue R250 for visualization of proteins or alternatively proteins were transferred onto 0.2 *μ*m nitrocellulose or PVDF membrane by Western blotting using a semidry blotter for 1 h at 100 mA. Membranes were blocked for 1 h in 3% dry milk (w/v) and 1% (w/v) bovine serum albumin (BSA) in PBS containing 0.05% (v/v) Tween 20. Blots were incubated with 1.0 *μ*g/mL polyclonal rabbit (Rb) anti-SLPI specific antibody (Abcam, Cambridge, UK), 1.0 *μ*g/mL polyclonal goat (Gt) anti-SLPI (Synergen Inc., Boulder, Colorado 80301) [21, 22], 1.0 *μ*g/mL polyclonal goat (Gt) anti-SLPI (R&D Systems), 0.2 *μ*g/mL polyclonal rabbit anti-myeloperoxidase (MPO), anti-hCAP-18, or anti-lactoferrin antibody (all purchased from Abcam, Cambridge, UK). Additional primary antibodies included 0.2 *μ*g/mL polyclonal goat antimatrix metalloprotease (MMP)-9 (R & D Systems), 1.0 *μ*g/mL monoclonal anti-talin-1, and 1.0 *μ*g/mL monoclonal anti-vinculin or monoclonal anti-actin antibody (Millipore). The secondary antibodies were HRP-linked anti-rabbit, -goat, or -mouse IgG (Cell Signalling Technology, Danvers, MA, USA). Immunoreactive protein bands were visualized employing SuperSignal West Pico Chemiluminescent Substrate (Pierce, Rockford, IL, USA) after exposure to Kodak X-Omat LS Film.

### 2.6. Neutrophil Electropermeabilization

Cell permeabilization was performed immediately before use of neutrophils as previously described [23, 24]. In brief, cells (5 × 10^6^) were washed in hypoosmolar buffer (Eppendorf, UK, Ltd.) and then resuspended in 400 *μ*L of ice-cold hypoosmolar buffer in the presence or absence of 1 *μ*M IP_3_. The cells were then transferred into an electroporation cuvette (2 mm gap, 400 *μ*L volume purchased from Eppendorf) and subjected to three discharges of 300 V using an Eppendorf Multiporator, with gentle stirring between the three pulses by pipetting [24]. Permeabilized cells were then incubated at room temperature for 5 min to allow incorporation of IP_3_ into electroporated neutrophils, immediately washed in PBSG, and then employed in chemotaxis assays or for Ca^2+^ flux measurements. Control experiments indicated that the cell permeabilization procedure did not affect cell viability as determined by trypan blue exclusion assays. 

### 2.7. Neutrophil Chemotaxis Assays

Chemotaxis assays were performed by measuring the percentage of neutrophils (2.5 × 10^5^cells/200 *μ*L) migrating towards IL-8 (1.2 nM) or fMLP (1 *μ*M) by employing a multiwell chemotaxis chamber (Neuro Probe, Inc., USA) and polyvinylprrolidone-free polycarbonate filters (10 *μ*m thick with 5 *μ*m pores). The stimulant was placed in the lower chamber (total volume of 90 *μ*L), and purified neutrophils were placed in the upper chamber (2.5 × 10^5^cells/200 *μ*L) with or without SLPI (0, 120, 240 or 480 nM). Neutrophil chemotaxis was quantified over 30 min at 37°C. The Neuroprobe chamber was then disassembled, and the polycarbonate filter was fixed with methanol, and cells were stained using Speedy-Diff solutions (Clin-tech Ltd, UK). The number of migrated neutrophils was determined microscopically employing a Nikon Eclipse TS100 microscope with 10 standardized × 400-high power fields counted for each well. For comparative analysis neutrophils treated with IL-8 or fMLP were set at a chemotactic index of 1, as indicated. Additional experiments were performed after electropermeabilization with 1 *μ*M IP_3_. Cells were then left unstimulated (control) or challenged with fMLP or IL-8. Data was represented as chemotactic index as previously described [25]. 

### 2.8. Measurement of Intracellular Ca^2+^ Flux

Measurement of intracellular cytosolic Ca^2+^ was performed employing the Fluo-4 NW Calcium Assay Kit (Invitrogen, Bio Sciences Ltd., Ireland) in the absence of extracellular Ca^2+^ according to the manufactures instructions. In brief, cells (2 × 10^5^/mL) were incubated for 30 min in dye loading solution containing the Ca^2+^ sensitive dye Fluo-4. Subsequently, cells were either left untreated or were exposed to fMLP (1 *μ*M) or IL-8 (1.2 nM) with changes in fluorescence recorded immediately upon addition of the stimulant. Fluorescence was recorded every 10 sec for 5 min employing a Victor X3 (PerkinElmer, Ireland) plate reader with excitation wavelength of 490 nm and emission at 535 nm. A subset of experiments was performed by incubating cells with either SLPI (480 nM), the PLC-*β* inhibitor U73122 (5 *μ*M), or the GPCR inhibitor pertussis toxin (500 ng/mL), prior to stimulation with fMLP (1 mM) or IL-8 (1.2 nM). Additionally, experiments were performed by pretreating cells with either SLPI (480 nM) or oxidised SLPI (480 nM), the later oxidised with 20 mM hydrogen peroxide (H_2_O_2_) as previously described [13]. 

### 2.9. Analysis of Neutrophil Degranulation and Secretion of SLPI

Neutrophils (5 × 10^6^/mL per reaction) suspended in PBSG remained untreated or preloaded with rhSLPI (480 nM) for 10 min, followed by fMLP/IL-8 (used in combination at 1 *μ*M and 1.2 nM, respectively, to ensure release of primary, secondary, and tertiary granules) stimulation for 10 or 20 min. Cell free supernatants were harvested following centrifugation at 500 ×g for 5 min at 4°C and analysed for degranulated proteins: MPO as a marker of primary granule release, hCAP-18 as a marker of secondary granule release, and MMP-9 as a marker of tertiary granule release, by Western blotting. In a subset of experiments the extracellular release of native cytosolic SLPI was analysed by Western blotting following cellular activation with PMA (1.6 *μ*M) for 0, 0.5, 1, or 10 min or fMLP (1 *μ*M) and IL-8 (1.2 nM) for 0, 10, 20, 30, 40, or 50 sec. Cell free supernatants were harvested as described above and acetone precipitated prior to SDS-PAGE and Western blot analysis for SLPI.

### 2.10. Neutrophil Elastase (NE) and Calpain Activity Assays

NE (34 nM) activity assays employing the specific substrate N-methoxysuccinyl-Ala-Ala-Pro-Val-p-nitroanilide as previously described [26]. Liberation of p-nitroaniline was measured at 405 nm at 37°C at 1 min intervals for 5 min. In additional studies the NE inhibitory activity of neutrophil cytosol was assessed before and after immunoprecipitation of SLPI. For immunoprecipitation experiments, neutrophil cytosols were obtained from 1 × 10^7^ cells and precleared by incubation with protein A-Sepharose beads for 1 h as previously described [18]. Goat polyclonal anti-SLPI (5 *μ*g) or isotype control IgG was then added to precleared cytosols for 1 h with rotation at 4°C. SLPI was then removed from the cytosol by incubation with reconstituted protein A-Sepharose beads for 1 h, and the anti-NE capacity of the resulting cytosol (25 *μ*g) was analysed. 

Kinetic analysis of calpain activity was determined using the Calpain-Glo protease assay (Promega Corporation, Madison, USA) which detects cleavage of the calpain substrate Suc-LLVY-aminoluciferin with luminescence recorded as per the manufacturer's instructions. The inhibitory effect of rhSLPI and calpastatin was analysed over a range of 0–640 nM and 0–80 nM, respectively. 

### 2.11. Inositol 1,4,5-Triphosphate Measurements

For analysis of IP_3_ we employed a recognised protocol and measured levels of IP_1_ which accumulates as a stable product of IP_3_ [27]. Neutrophils (1 × 10^5^) remained untreated or were preloaded with SLPI (480 nM) as already described. As positive controls the PLC-*β* inhibitor U73122 (5 *μ*M) and the GPCR inhibitor pertussis toxin (500 ng/mL) were employed prior to stimulation with fMLP (1 *μ*M) or IL-8 (1.2 nM) for 10 sec. Cells were then lysed, and intracellular IP_1_ levels were determined by use of an IP-One HTRF assay kit in a final reaction volume of 200 *μ*L as per the manufacturer's instructions. 

### 2.12. Flow Cytometry Analysis

Flow cytometry was carried out to evaluate the membrane expression of CD16b as a measure of cell purity [18] or CD66b (secondary and tertiary granules) and CD63 (primary granule) as a measure of degranulation [24]. Cells remained untreated or were stimulated with fMLP (1 *μ*M) and IL-8 (1.2 nM) for 10 min at 37°C. Neutrophils were then fixed (4% (w/v) paraformaldehyde) and blocked (2% (w/v) BSA) for 30 min at room temperature. After washing (PBS × 2) neutrophils (1 × 10^6^) were incubated with 1 *μ*g/100 *μ*L of mouse monoclonal anti-CD16b (Santa Cruz, Germany), FITC labelled anti-CD66b, or PE labelled anti-CD63 (both from BD Bioscience, UK). Control samples were exposed to relevant nonspecific isotype control IgG or secondary labeled antibody alone (FITC-labelled bovine anti-mouse). In additional experiments the ability of fMLP to interact with its cognate receptors on the neutrophil membrane in the presence of rhSLPI was performed as previously described [28]. In brief, purified neutrophils (1 × 10^7^/mL) were left untreated or exposed to fMLP (10 *μ*M) or rhSLPI (480 nM) for 1 min, followed by incubation with FITC-labeled fMLP (1 *μ*M) for 1 min. Cells were washed in PBS and fluorescence was counted using a BD FACSCalibur flow cytometer (BD Bioscience, Germany) with a total of 10,000 events acquired. The data were analysed and the mean fluorescence intensity (MFI) for each experiment was determined using BD CellQuest Pro software. 

### 2.13. Confocal Immunofluorescence

Adherent neutrophils were produced by placing neutrophils in PBS on Polysine microscope slides (AGB Scientific Ltd., Ireland) after which they were incubated at 37°C for 5 min and then for a further 10 min at 37°C in the presence of fMLP (1 *μ*M) and IL-8 (1.2 nM) or PMA (1.6 *μ*M). Cells were then fixed with 4% (w/v) paraformaldehyde in PBS for 10 min and permeabilised with 0.2% (v/v) Triton X-100 in PBS for 10 min. Subsequently cells were blocked for 1 h with 1% (w/v) BSA in PBS and then incubated with 1 *μ*g/mL FITC-labeled rabbit polyclonal anti-SLPI for 45 min and washed in PBS. Cells were mounted employing VECTASHIELD mounting medium with DAPI for nuclear staining (Vectashield Lab, UK). Control samples were exposed to non-specific FITC-labeled isotype control IgG. All immunofluorescence slides were viewed and images were acquired using a Zeiss LSM710 confocal immunofluorescence microscope.

### 2.14. Statistical Analysis

The data was analysed with the GraphPad Prism version 4.03 for Windows (GraphPad Software, San Diego, CA, USA) and results were expressed as mean ± standard error (S.E.) of the mean. The Mann-Whitney *U*-test or analysis of variance (ANOVA) sample tests followed by Bonferri correction were employed to identify significant differences. Experiments were performed in triplicate, and a *P*-value < 0.05 was deemed significantly different. 

## 3. Results and Discussion

### 3.1. The Immunomodulatory Effects of Cytosolic SLPI on Neutrophil Migration and Degranulation

SLPI has been previously reported to exert an anti-inflammatory effect on immune cell function [20, 29], and in this respect SLPI has been shown to inhibit monocytic CD4 lymphocyte proliferation and Th1 cytokine (INF-*γ*) release [30]. Indeed, T lymphocytes play a key role in the pathogenesis of CF [31] and COPD [32] lung disease and have been identified as the predominant cell type in subepithelial bronchial tissue of CF patients [31]. Th1, Th2, and Th17 cells may augment the cytokine/chemokine profile in the airways of CF patients thus contributing to the chronic recruitment of neutrophils [31]. Thus the objective of this study was to investigate whether SLPI exerted an anti-inflammatory effect on neutrophil function, and for this reason it was first necessary to confirm its cellular localisation.

**Figure 1:**
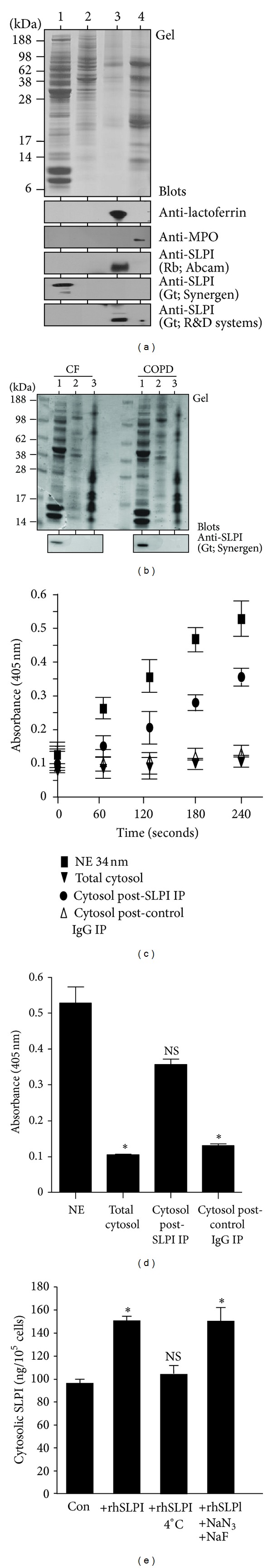
Localization and activity of native SLPI in peripheral blood neutrophils. (a) Coomassie blue stained gel of isolated neutrophils subjected to subcellular fractionation yielding a cytosol fraction (lane 1), membrane fraction (lane 2), and secondary and primary granule fractions, lane 3 and 4 respectively. Western blotting employed polyclonal rabbit (Rb) or goat (Gt) antibody to SLPI, which localized SLPI to both the cytosolic and secondary granule fractions. As controls, MPO and lactoferrin were detected using rabbit polyclonal antibodies as markers for primary and secondary granules, respectively. (b) Coomassie blue stained gel of isolated CF and COPD neutrophils subjected to subcellular fractionation yielding a cytosol fraction (lane 1), membrane fraction (lane 2), and pooled primary and secondary granule fractions (lane 3). Western blotting employed polyclonal Rb antibody confirming cytosolic localization of SLPI in patient samples. (c) The NE inhibitory activity of neutrophil cytosol was assessed before and after immunoprecipitation (IP) of SLPI using Gt polyclonal anti-SLPI antibody (Synergen). The kinetics of inhibition is illustrated in (c), and data of the final time point (240 sec) is plotted in (d). Control experiments included isotype control Gt IgG. *P < 0.05 between NE control. (e) Addition of 480 nM rhSLPI to cells (2 × 10^5^/mL) increased the concentration of cytosolic SLPI by approximately 50% above the untreated cells (Con), as determined by ELISA. Experiments were repeated at 4°C or in the presence of NaN_3_ (15 mM) and NaF (10 mM). *P < 0.05 between untreated cells (con). Results illustrated in (a) and (b) are representative gels and blots of 3 separate experiments. Results illustrated in panel (c)–(e) were performed in triplicate and each bar is the mean ± S.E. (NS, no significant difference, *P < 0.05 calculated by Student's *t*-test).

Localisation of SLPI within many cell types known to produce this protein is still being elucidated. For example, it has been reported that cellular SLPI is localised in the nuclei of bronchial epithelial cells exposed to 17*β*-estradiol resulting in NF-*κ*B inhibition and IL-8 gene expression [33]. SLPI has also been detected in the nuclei of alveolar macrophages in patients with pulmonary sepsis, but not in healthy controls [20], indicating that the localisation of SLPI in disease states may differ from healthy control cells. Within the present study by subcellular fractionation of resting neutrophils and Western blot analysis employing both rabbit and goat polyclonal anti-SLPI antibodies, SLPI was identified as a component of the cell cytosol and secondary granule fractions (co-localising with lactoferrin) and was absent from unstimulated membranes and primary granule fractions containing MPO (Figure 1(a)). Localization of SLPI to these compartments is in keeping with previously published data [11, 16]. As the role of cytosolic SLPI was the focus of ensuing experiments, we also confirmed the presence of SLPI in cytosolic fractions of CF and COPD cells (Figure 1(b)). 

Having identified the position of SLPI in the neutrophil further experiments were designed to establish whether cytosolic SLPI possessed antiprotease activity. Results of anti-NE kinetic measurements indicated that cytosolic SLPI contained anti-NE capacity (Figure 1(c)) as both the rate and total level of NE catalytic activity (34 nM) were negated by freshly isolated neutrophil cytosol (25 *μ*g/10 *μ*L); an effect significantly reversed in samples in which SLPI was extracted by immunoprecipitation (Figure 1(c)). At the 240 sec time point (4 min), cytosol that had been subjected to immunoprecipitation for SLPI removal was 66.7 ± 2.1% less active in inhibiting NE as compared with complete cytosol (*P* < 0.05) and illustrated no significant difference compared to the NE control reaction (Figure 1(d)). 

To begin to understand the role of intracellular SLPI in neutrophil cell function our approach was to increase the level of this anti-protease within the cell cytosol. To achieve this, cells were resuspended in PBSG containing exogenous rhSLPI for 10 min and then washed to remove protein that had not been taken up by the cells. To confirm that rhSLPI was internalized, the concentration of SLPI in neutrophil cytosolic fractions was determined by ELISA. Results revealed that addition of 480 nM rhSLPI significantly raised the concentration of cytosolic SLPI from 96 ± 6.3 ng to 151 ± 4.8 ng per 1 × 10^5^ cells (*P* < 0.05, *n* = 6) indicating permeability of the cell membrane to exogenous rhSLPI (Figure 1(e)). To examine internalization of rhSLPI in further detail experiments were repeated at 4°C, thereby applying a temperature block to inhibit transport processes [20]. Under these reduced temperature conditions results indicated that the uptake of SLPI and localization to the cell cytosol were significantly inhibited by 30.2 ± 3.4% (Figure 1(e)). The role of endocytosis was also assessed by pretreating cells with the endocytosis inhibitors NaN_3_ (15 mM) and NaF (10 mM) [20]. These inhibitors had no significant effect on rhSLPI uptake and cytosolic association (Figure 1(e)). Collectively, these results indicate that exogenously added rhSLPI is taken up by the cell and is localized in the cytosol and is not contained within endosomes. These results are in line with previously published data on the internalization of rhSLPI by mononuclear cells [20]. In this later publication exogenously added rhSLPI was localized to the cytosol and nucleus of U937 monocytes. SLPI has been characterised as an arginine rich cationic molecule [34], and it has been reported that the positively charged nature of the protein facilitates its transduction across negatively charged membranes [20, 34]. Moreover, investigation of the anti-HIV-1 inhibitory activity of SLPI has prompted the identification of membrane proteins that are capable of binding to SLPI. In this regard, SLPI has been shown to interact with the phospholipid binding protein annexin II on the surface of human macrophage cell membranes [35] and has also been described as a ligand for phospholipid scramblases 1 and 4 (PLSCR1 and PLSCR4) [36]. This later interaction suggested that SLPI disrupts interplay between PLSCR1 and the CD4 receptor on the surface of CD4^+^T lymphocytes thus preventing HIV-1 viral infection [36].

**Figure 2:**
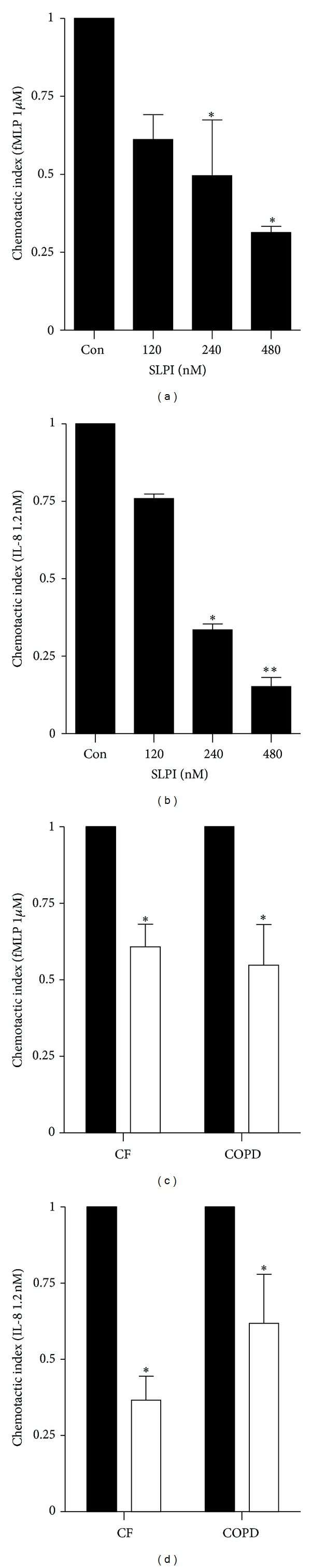
rhSLPI inhibits neutrophil chemotaxis. (a) and (b) An increase in chemotactic inhibition efficiency of increasing concentrations of SLPI (120, 240, and 480 nM). The chemotactic index represents the ratio of neutrophils that migrated towards fMLP (1 *μ*M) or IL-8 (1.2 nM) over 30 min at 37°C. (c) and (d) fMLP and IL-8-induced mean chemotactic index of neutrophils (2.5 × 10^5^cells/200 *μ*L) isolated from individuals with CF (*n* = 5) or individuals with COPD (*n* = 5), in the presence (□) or absence (■) of 480 nM SLPI. Results illustrated were performed in triplicate and each bar is the mean ± S.E. *P < 0.05 or **P < 0.01 versus untreated control cells (Con) with statistical significance calculated by Student's *t*-test.

Within the present study subsequent experiments investigated the effect of cytosolic SLPI on directional chemotaxis, a fundamental neutrophil cellular response in terms of pulmonary inflammation. Cells (2.5 × 10^5^/200 *μ*L) were first preloaded with exogenous rhSLPI (0, 120, 240 or 480 nM), washed, and then exposed to IL-8 (1.2 nM) or fMLP (1 *μ*M), two stimuli with known effective chemoattractant properties [37, 38]. As shown in Figures 2(a) and 2(b), rhSLPI inhibited both IL-8 and fMLP-induced neutrophil directional chemotaxis in a dose dependent manner with an IC_50_ of approximately 240 nM recorded for both stimuli. Excessive neutrophil infiltration to the lung is a key pathogenic feature for disease progression in individuals with CF and COPD. For this reason we examined the effect of rhSLPI on CF and COPD neutrophil chemotaxis in response to inflammatory stimuli *in vitro*. Results confirmed the inhibitory action on cell migration and demonstrated that cells preloaded with rhSLPI showed a markedly reduced capacity to migrate in response to fMLP and IL-8 (Figures 2(d) and 2(c), respectively; *P* < 0.05, *n* = 5). An IC_50_ of 611 nM and 376 nM for CF cells and an IC_50_ of 542 nM and 627 nM for COPD cells were recorded for fMLP and IL-8 stimulation, respectively. Thus a greater level of SLPI was required to inhibit CF and COPD neutrophil chemotaxis compared to control cells. This latter result may be a consequence of neutrophil priming within the circulation of individuals with CF and COPD due to persistent inflammation. Indeed, studies have shown that CF neutrophils are primed and unresponsive to IL-10 induced anti-inflammatory signals [39]. *Pseudomonas *alginate [40], TNF-*α*, and IL-8 have been shown to be important priming agents for CF neutrophils, causing greater release of MPO [41] and NE, compared to neutrophils from control subjects and individuals with bronchiectasis [42].

**Figure 3:**
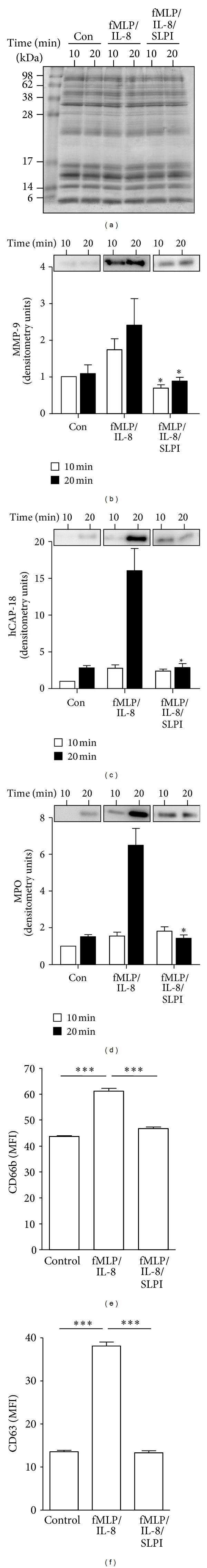
rhSLPI inhibits neutrophil degranulation. Neutrophils (5 × 10^6^/mL) isolated from healthy individuals were either untreated (Con) or exposed to SLPI (480 nM), followed by stimulation with fMLP (1 *μ*M) and IL-8 (1.2 nM) at 37°C. The use of equal cell numbers in each reaction is demonstrated by the identical electrophoretic profile of whole cell lysates in the Coomassie blue stained gel (a). Cell free supernatants were collected at 10 (□) or 20 min (■) and Western blotted for markers of tertiary ((b), MMP-9), secondary ((c), hCAP-18), or primary granule release ((d), MPO). (e) and (f), Healthy control neutrophils were fixed in 4% (w/v) paraformaldehyde following 10 min IL-8/fMLP stimulation in the presence or absence of SLPI (480 nM) and analysed by flow cytometry employing a FITC-labeled CD66b (e) or CD63 antibody (f). Data are represented as mean fluorescent intensity (MFI). All results (expressed as relative densitometry units) were performed in triplicate, and each bar is the mean ± S.E. A representative Western blot is illustrated. *P < 0.05 between SLPI treated and untreated at the respective time points calculated by Student's *t*-test.

The degranulation of proteolytic enzymes and peptides from the neutrophil upon activation is a tightly regulated process in order to prevent unnecessary damage to tissues [43]. For this reason we evaluated the inhibitory effect of rhSLPI on the kinetics of degranulation. In this experiment we used fMLP (1 *μ*M) and IL-8 (1.2 nM) in combination to ensure exocytosis of all three neutrophil granule types (primary, secondary, and tertiary), and release of granule proteins was quantified in the extracellular supernatant by immunoblotting. The use of equal cell numbers (5 × 10^6^/mL) in each reaction is demonstrated by identical Coomassie blue stained electrophoretic profiles of whole cell lysates prepared form cells employed in each reaction (Figure 3(a)). Levels of cell released MMP-9 from tertiary granules (Figure 3(b)), hCAP-18 from secondary granules (Figure 3(c)), and MPO from primary granules (Figure 3(d)) were significantly reduced post 20 min stimulation in cells preloaded with 480 nM rhSLPI (a 60%, 83%, and 80% reduction respectively, *P* < 0.05). As tertiary granules are more readily discharged, the inhibitory action of rhSLPI was apparent at the 10 min time point (70% reduction, *P* < 0.05). As an alternative approach we investigated the membrane expression of CD66b and CD63. CD66b is a membrane receptor that is exclusively expressed on the membrane of secondary and tertiary granules, and CD63 is present on primary granule membranes [24]. Upon degranulation both CD66b and CD63 become expressed on the cell surface. As illustrated in Figures 3(e) and 3(f), membrane expression of CD66b and CD63 increased by 40% and 180%, respectively, after 10 min fMLP/IL-8 combined stimulation compared to unstimulated cells (*P* < 0.001). In contrast, upregulation of CD66b and CD63 to the plasma membrane was significantly decreased in stimulated neutrophils preloaded with SLPI compared to cells unexposed to SLPI (*P* < 0.001). Collectively, this data successfully demonstrates the inhibitory effect of rhSLPI on neutrophil chemotaxis and degranulation via IL-8 and fMLP GPCR signalling. 

### 3.2. Characterisation of the Mode of Action of SLPI

**Figure 4:**
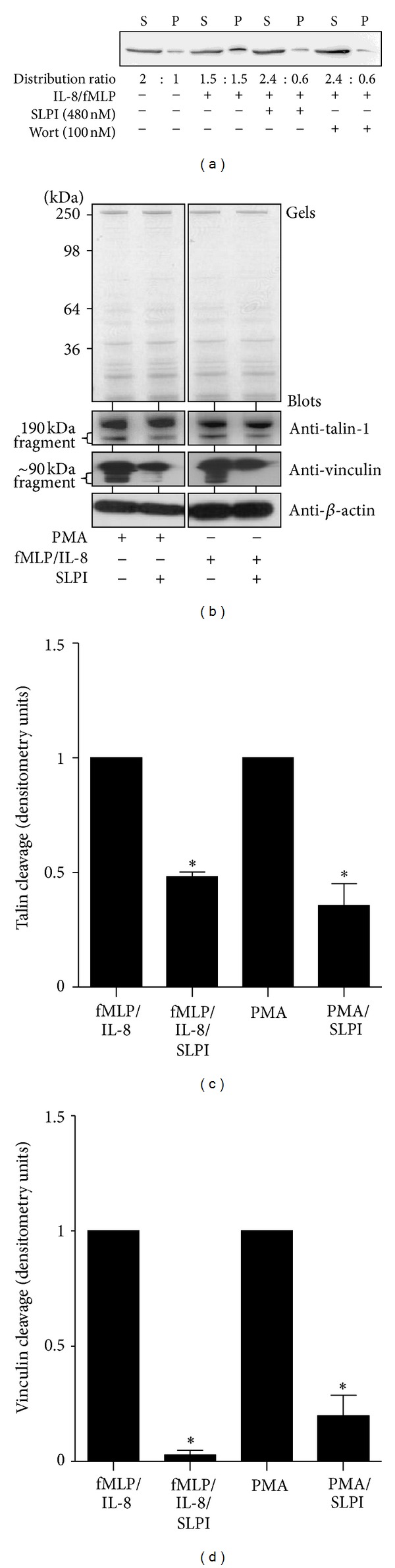
SLPI inhibits neutrophil cytoskeletal rearrangements. (a) Immunoblot with anti-actin antibody for the distribution of G-actin (in the supernatant fraction, S) and F-actin (in the pellet fraction, P) in untreated cells, IL-8/fMLP (1.2 nM/1 *μ*M), SLPI (480 nM), or control wortmannin (Wort; 100 nM) treated cells. The distribution ratios calculated using constants obtained of Western blot densitometry values are illustrated. (b) The effect of SLPI (480 nM) on talin-1 (190 kDa fragment) or vinculin cleavage (90 kDa fragment) after PMA (1.6 *μ*M, positive control), or IL-8/fMLP activation for 10 min was analysed by Western blotting using anti-talin-1 and anti-vinculin monoclonal antibodies. Equal sample loading was confirmed by Coomassie blue stained gels (top panels) and the *β*-actin immunoblots (lower panels). Results of three separate experiments were expressed as relative densitometry units ((c) and (d)) and each bar is the mean ± S.E. *P < 0.05 between SLPI treated and untreated cells for the respective stimuli calculated by Student's *t*-test.

Remodelling of the actin cytoskeleton is a prerequisite for cell chemotaxis and is a crucial event in the degranulation process. To study the effect of preloading cells with rhSLPI on the redistribution of F-versus G-actin after IL-8/fMLP exposure, a differential centrifugation assay was employed to analyse *in situ* F-actin levels. Preloading of cells with rhSLPI (480 nM) or exposure to wortmannin (100 nM) as a control [18] suppressed the IL-8/fMLP-induced change in the ratio of G-actin (supernatant fraction) versus F-actin (pellet fraction) (Figure 4(a)). In addition, as the C-terminal fragment of talin (190 kDa) is an actin nucleating protein which binds to G-actin and also the fact that cleavage of talin-1 is critical to focal adhesion disassembly [44], we investigated the possibility that SLPI regulates neutrophil chemotaxis by affecting talin cleavage. Neutrophils which were either untreated or preloaded with rhSLPI (480 nM/1 × 10^7^ cells) were stimulated with PMA (positive control) or fMLP/IL-8 for 10 min, and the level of talin-1 cleavage was assessed by immunoblotting using a mouse monoclonal anti-talin-1 antibody (Figure 4(b)). Site-specific cleavage of talin-1 yielding a 190 kDa fragment was observed in fMLP/IL-8 and PMA activated whole cell lysates, an effect consistently inhibited by preloading cells with rhSLPI (50% and 64% inhibition, resp.), (Figure 4(c), *P* < 0.05). Moreover, we evaluated the effect of rhSLPI on vinculin cleavage, a protein involved in neutrophil adhesion and pseudopod formation [45]. Results revealed that vinculin cleavage in response to fMLP/IL-8 and PMA was significantly reduced in cells preloaded with rhSLPI, as demonstrated by Western blot analysis (Figure 4(b)) and densitometric quantification (96% and 84% inhibition, respectively, Figure 4(d)). 

The observation that rhSLPI could inhibit talin and vinculin cleavage prompted us to initially investigate the hypothesis that SLPI inhibited the Ca^2+^ dependent neutral cysteine protease activity of calpain-1 and calpain-2, which play an important role in cell migration by cleaving talin and vinculin [46]. SLPI has been shown to inhibit a broad range of serine proteases [47], but, unlike the antiprotease alpha-1 antitrypsin (AAT), SLPI has not been shown to inhibit other protease classes. For example, AAT has been shown to inhibit caspase-3 activity [48] and to modulate metalloprotease ADAM-17 activity, thereby regulating neutrophil chemotaxis in response to soluble immune complexes [18]. However, unlike AAT or the natural inhibitor calpastatin, which have been shown to inhibit neutrophil calpain [49], the results of the present study demonstrate that SLPI had no direct effect on calpain-1 or calpain-2 activity (see Supplementary Figure  1 in Supplementary Material available online on http://dx.doi.org/10.1155/2013/560141).

**Figure 5:**
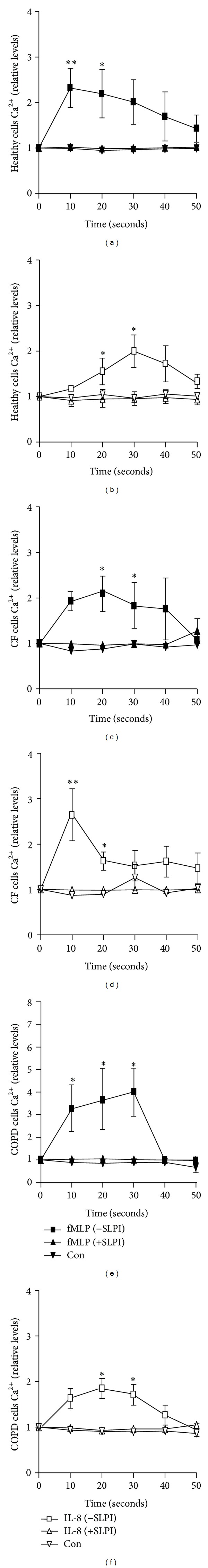
SLPI inhibits intracellular Ca^2+^ flux. Neutrophils (2 × 10^5^/mL) were isolated from healthy donors ((a) and (b)), individuals with CF ((c) and (d)) or COPD ((e) and (f)) (*n* = 5 for each cohort). Cells remained untreated (control, con) or treated with 480 nM SLPI for 5 min prior to stimulation with fMLP (1 *μ*M) or IL-8 (1.2 nM) for 10, 20, 30, 40, or 50 sec. Intracellular cytosolic Ca^2+^ was analysed using the Invitrogen Fluo-4NW Calcium Assay kit. Each point is the mean ± S.E. (*n* = 5, *P < 0.05 and **P < 0.001 between untreated and SLPI treated cells calculated by 2 way ANOVA followed by Bonferroni test).

Upon activation of neutrophils in the absence of extracellular Ca^2+^, the rise in cytosolic Ca^2+^ levels occurs by release from intracellular sites. As a result of the rise in Ca^2+^, the neutrophil initiates cytoskeletal rearrangements required for degranulation and chemotaxis [50]. Therefore, we next explored the possibility that SLPI inhibits activation of neutrophils by regulating Ca^2+^ mobilisation from intracellular stores. Preloading neutrophils (2 × 10^5^/mL) isolated from healthy individuals with rhSLPI (480 nM) in a Ca^2+^ free buffer significantly inhibited the rise in cytosolic Ca^2+^ levels from intracellular stores triggered by fMLP (Figure 5(a), *P* < 0.01 at 10 sec and *P* < 0.05 at 20 sec) and IL-8 (Figure 5(b), *P* < 0.05 at 20 and 30 sec) compared to untreated control cells. Of note, the observed spike in Ca^2+^ flux in healthy control cells not treated with SLPI is consistent with previously published data [18]. Furthermore, neutrophils isolated from CF stable individuals preloaded with rhSLPI also failed to initiate a Ca^2+^ spike upon stimulation with fMLP (Figure 5(c), *P* < 0.05 at 10 and 20 sec) or IL-8 (Figure 5(d), *P* < 0.01 and *P* < 0.05 at 10 and 20 sec, resp.). Additionally, when Ca^2+^ levels were analysed in neutrophils isolated from individuals during an acute exacerbation of COPD, there was a statistically significant decrease in the cytosolic Ca^2+^ levels of fMLP and IL-8 stimulated cells preloaded with rhSLPI (480 nM) compared to untreated cells (*P* < 0.05 at 10 and 20 sec, Figures 5(e) and 5(f)). Taken together, these results indicate that cytosolic SLPI inhibits Ca^2+^ flux in cells from patients or healthy donors when exposed to proinflammatory stimuli.

**Figure 6:**
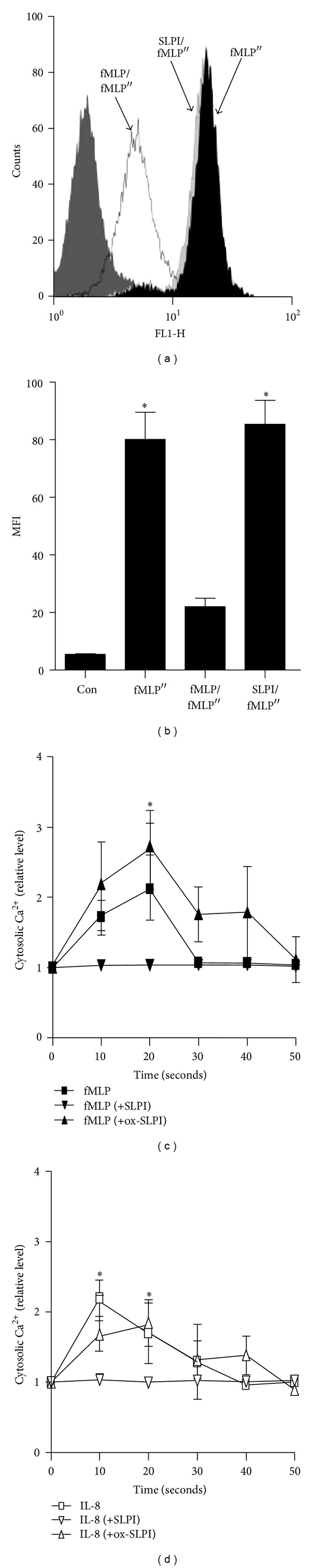
Intracellular nonoxidised SLPI exerts immunomodulatory activity. (a) Neutrophils (1 × 10^7^/mL) were exposed to unlabeled fMLP (10 *μ*M) or SLPI (480 nM) for 1 min, followed by FITC-labeled fMLP (fMLP′′) (1 *μ*M), and the level of bound fMLP′′, quantified by FACS. The negative control (unlabeled cells) is illustrated in dark grey and a total of 10,000 events were collected. (b) Results in mean fluorescence intensity units (MFI) demonstrate that pre-incubation with unlabeled fMLP, but not rhSLPI, prevented binding of fMLP′′ to the cell membrane (*P < 0.05 compared to fMLP/fMLP′′ cells). (c) and (d); Neutrophils (1 × 10^7^/mL) remained untreated or treated with 480 nM SLPI or 480 nM oxidised SLPI (ox-SLPI) for 5 min, prior to stimulation with fMLP ((c), 1 *μ*M) or IL-8 ((d), 1.2 nM). Ox-SLPI did not inhibit the fMLP or IL-8 induced Ca^2+^ flux (*P < 0.05 between SLPI and ox-SLPI). Each experiment was performed in triplicate, each point is the mean ± S.E. and statistical significance was calculated by 2-way ANOVA followed by Bonferroni test or Student's *t*-test.

 To rule out the possibility that SLPI inhibits Ca^2+^ flux in cells by preventing IL-8 and fMLP interacting with their respective receptors, studies on agonist-receptor interactions were performed. Whilst exposure to FITC-labeled fMLP resulted in specific binding of FITC-fMLP on neutrophil membranes by flow cytometry (Figures 6(a) and 6(b), pre-exposure of cells (1 × 10^7^/mL) to unlabeled fMLP prevented FITC-fMLP binding thereby reducing the mean florescence intensity reading by approximately 75% (40.05 ± 4.7 to 11.04 ± 1.45 MFI, Figures 6(a) and 6(b)). In contrast, pre-loading of neutrophils with rhSLPI (480 nM) had no effect on the FITC-fMLP fluorescence reading (42.67 ± 4.164 compared to 40.05 ± 4.471 in the presence of SLPI), indicating that the immuno-regulator activity of SLPI was not a result of inhibiting fMLP binding to the cell membrane. Additionally, results indicated that preloading cells with rhSLPI did not impair IL-8 membrane binding (results not shown). Moreover, reaction with H_2_O_2_, a major oxidant generated by activated neutrophils, oxidizes all four methionine residues in SLPI, resulting in substantial diminution of its NE inhibitory activity [13]. In the present study, oxidised rhSLPI possessed decreased inhibitory capacity over intracellular Ca^2+^ flux upon activation with fMLP (1 *μ*M) or IL-8 (1.2 nM, Figures 6(c), 6(b), and 6(d)) suggesting the requirement of the active site of SLPI for the observed inhibitory effect on Ca^2+^ flux. 

**Figure 7:**
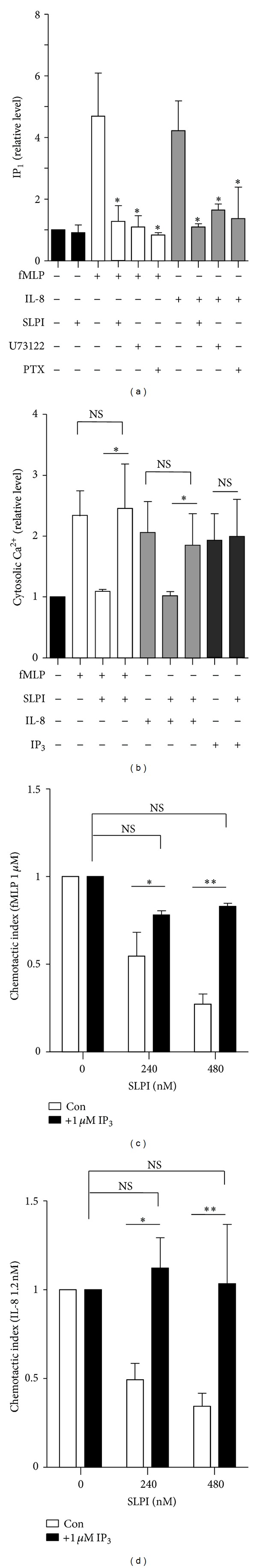
SLPI inhibits production of IP_3_. (a) Preloading neutrophils (1 × 10^5^/mL) with SLPI (480 nM) significantly reduced the accumulative levels of IP_1_ induced by fMLP (1 *μ*M) or IL-8 (1.2 nM) activation (final reaction volume of 200 *μ*L). Positive controls included U73122 (5 *μ*M) and pertussis toxin (PTX, 500 ng/mL). (b) Cells remained untreated or preloaded with SLPI (480 nM), electropermeabilized in the presence or absence of 1 *μ*M IP_3_ and then stimulated with fMLP (1 *μ*M) or IL-8 (1.2 nM) for 10 sec. (c) and (d) Neutrophils were isolated from healthy individuals and the effect of SLPI (240 or 480 nM) on the cell chemotactic index induced by fMLP (1 *μ*M) or IL-8 (1.2 nM) was determined in the presence (■) or absence (con, □) of 1 *μ*M IP_3_. SLPI had no significant inhibitory effect on neutrophil chemotaxis in the presence of augmented cytosolic IP_3_. Each point is the mean ± S.E. (*n* = 5, NS: no significant difference, and *P < 0.05 and **P < 0.001 calculated by Student's *t*-test).

In response to either fMLP or IL-8 the initial rise in intracellular cytosolic Ca^2+^ requires IP_3_ production, and subsequent IP_3_ occupancy of receptors on calciosomes induces a rapid release of Ca^2+^ [51, 52]. Therefore, we next explored the possibility that cytosolic rhSLPI inhibits Ca^2+^ cytosolic flux by preventing upstream IP_3_ production. For this analysis we measured levels of IP_1_ which accumulates as a stable product of IP_3_ [27]. Pre-loading of neutrophils (1 × 10^5^/mL) with rhSLPI (480 nM) significantly reduced cumulative levels of IP_1_ upon fMLP or IL-8 activation (an approximate 75% reduction for both stimuli, *P* < 0.05), with similar levels to unstimulated cells observed (Figure 7(a)). Positive controls for this experiment included addition of the PLC-*β* inhibitor U73122 (5 *μ*M) or the GPCR inhibitor pertussis toxin (PTX, 500 ng/mL), with significant inhibition of IP_1_ accumulation observed for both (*P* < 0.05). Substantiating this result, it was observed that the inhibitory effect of rhSLPI (480 nM) on Ca^2+^ flux induced by fMLP and IL-8 was overcome by augmenting cytosolic levels of IP_3_ (1 *μ*M) (Figure 7(b)). For this latter experiment IP_3_ was incorporated into electroporated neutrophils and the cells immediately employed in Ca^2+^ flux measurements. Moreover, results revealed that rhSLPI employed at a concentration of either 240 or 480 nM was unable to reduce the chemotactic index of cells loaded with IP_3_ in response to either fMLP (Figure 7(c)) or IL-8 (Figure 7(d)). 

Collectively, these results confirm that cytosolic SLPI did not impede agonist receptor interaction but successfully modulated IP_3_ production and subsequent release of Ca^2+^ from IP_3_-regulated internal stores. However, as the production of IP_3_ is a direct outcome of PLC-*β* activation, further experiments are required to fully understand how SLPI modulates PLC-*β* activation. Moreover, it is possible that SLPI affects Ca^2+^ flux via modulation of other immune signalling pathways including the mitogen-activated protein kinase (MAPK) cascade, ERK1/2 and p38 MAPK [53–55], modulation of the increase in intracellular Ca^2+^ associated with activation of the transcription factor NF-*κ*B [56], or by regulation of cAMP-dependent protein kinase A [3]. Nevertheless, the maintenance of intracellular Ca^2+^ levels via IP_3_ modulation represents a novel therapeutic strategy in inflammatory conditions such as COPD and CF, where neutrophil infiltration to the airways causes excessive tissue damage. In line with this theory, U73122, a membrane permeable aminosteroid PLC inhibitor, which we have been used as a positive control in this study, has previously been shown in an *in vivo* animal model to significantly inhibit neutrophil infiltration to the peritoneal cavity upon LPS injection, in addition to reducing IL-8 and leukotriene B_4_ induced Ca^2+^ flux in neutrophils [57]. The findings of the present study are highly relevant in the consideration of rhSLPI as a modulator of Ca^2+^ flux and therapeutic modality in chronic neutrophilic airway inflammatory disorders. In support of this concept applications of aerosolized anti-proteases such as SLPI have been investigated as potential therapeutics for people with CF and COPD [58, 59]. Aerosolized rhSLPI (100 mg, twice daily for 1 week) has previously been administered to individuals with CF [59] with results revealing increased epithelial lining fluid levels of SLPI and significantly reduced levels of active NE (pretreatment 11.1 ± 1.8 *μ*M NE to posttreatment 6.1 ± 1.2 *μ*M NE). A further *in vivo* study carried out in rats reported that rhSLPI could remain biologically active in the lungs for at least 8 h [60]. When administered intratracheally, (8.6 mg/kg) the reported half-life of SLPI was 4 to 5 h, indicating minimal metabolism in the airways. rhSLPI has also been administered intravenously to sheep [21]. In this latter study, following an infusion of rhSLPI (1 g) over 10 min the half-life of rhSLPI was reported as 1.8 h; however if the rate of infusion was slowed, SLPI excretion was significantly decreased with a 3 h infusion associated with 9% excretion [21]. Intact SLPI was detected in lymph and epithelial lining fluid, which displayed anti-NE capacity in line with the levels of SLPI [21].

### 3.3. Secretion of SLPI from the Neutrophil Coincides with Cell Activation

**Figure 8:**
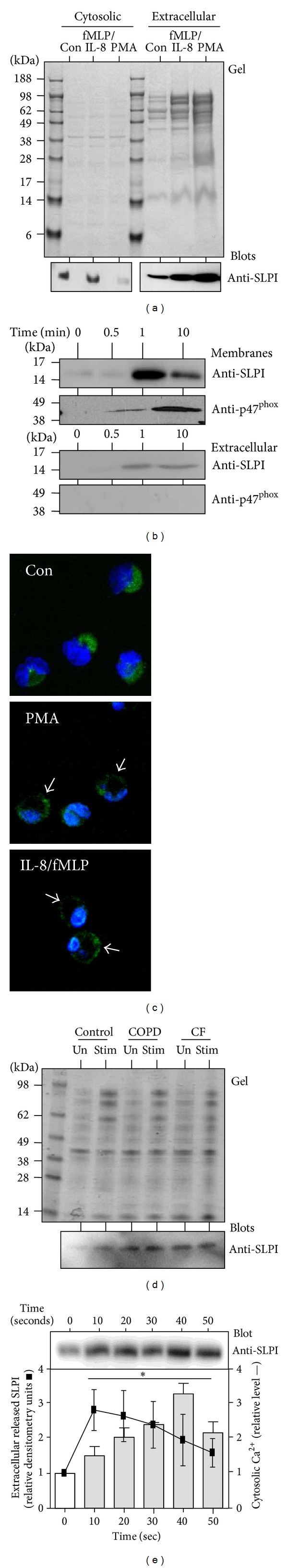
SLPI is secreted from the cell upon activation. (a) Coomassie Blue stained gel (top panel) and immunoblots (bottom 2 panels) employing anti-SLPI antibody (Gt, Synergen) for its respective distribution (intracellular or extracellular) in control unstimulated cells (Con) and after IL-8 (1.2 nM) and fMLP (1 *μ*M) or PMA (1.6 *μ*M) activation for 10 min. (b) Donor neutrophils (5 × 10^6^/mL) were stimulated with PMA (1.6 *μ*M) for 0, 0.5, 1, or 10 min before sonication and preparation of membrane (top panels) and extracellular released protein fractions (bottom panels). SLPI and p47^phox^ translocation was analysed by Western blotting employing polyclonal rabbit antibodies. (c) The distribution of SLPI in resting unstimulated cells (Con) and after PMA or fMLP (1 *μ*M) and IL-8 (1.2 nM) activation for 10 min was detected using an FITC labeled rabbit polyclonal anti-SLPI antibody (green fluorescence). The distribution of SLPI was predominantly cytosolic in unstimulated cells, and after stimulation condensed around the margin of the cell (indicated by white arrow). DAPI stained nuclei are represented in blue (×40 magnification, ×10 zoom). (d) Coomassie blue stained gel (top panel) and immunoblot (bottom panel) employing rabbit anti-SLPI antibody for detection of released SLPI in the extracellular supernatants of unstimulated (Un) or fMLP/IL-8 stimulated (Stim) healthy control (Con), COPD or CF cells (5 × 10^6^/mL). (e) Neutrophils isolated from healthy donors were incubated at 37°C and were treated with fMLP/IL-8. Cell free supernatants were collected at 0, 10, 20, 30, 40 or 50 sec and Western blotted for cell secreted SLPI (top panel) and intracellular cytosolic Ca^2+^ was analysed using the Invitrogen Fluo-4 Calcium Assay kit. Results of three separate experiments were expressed as relative densitometry units (bar graph), and each bar is the mean ± S.E. *P < 0.05 is between 0 time point and statistical significance was calculated by Student's *t*-test.

The question next investigated was how cells overcome the inhibitory effect of native cytosolic SLPI thereby leading to Ca^2+^ flux in neutrophils. Indeed, neutrophils have previously been shown to secrete SLPI [11], and for this reason ensuing experiments were designed to determine whether the kinetics of SLPI release upon cell activation corresponded to the spike of Ca^2+^ flux. By Western blotting, it was found that neutrophils (5 × 10^6^/mL) secrete cytosolic SLPI to the outside of the cell in response to both IL-8 (1.2 nM) and fMLP (1 *μ*M) or PMA (1.6 *μ*M) activation for 10 min (Figure 8(a)). A reduction in the level of cytosolic SLPI was observed, most prominently after PMA activation, with a concomitant increase in the level of extracellular SLPI detected. These results are in keeping with a previous study that recorded significant amounts of SLPI secreted from activated neutrophils (3 *μ*g/10^6^ cells/24 h) as compared with an epithelial and type II pneumocyte cell line [11]. This set of experiments were expanded and included subcellular fractionation and isolation of membranes following cell (5 × 10^6^/mL) activation with PMA (1.6 *μ*M) at 0, 0.5, 1, and 10 min. Results revealed that over the timecourse explored, progressively higher quantities of SLPI became associated with the plasma membrane (Figure 8(b) upper panel) followed by sequential secretion to the outside of the cell (Figure 8(b) lower panel). Translocation of the NADPH oxidase component p47^phox^ from the cytosol to the membrane upon cell activation was used as a positive control [61]; however p47^phox^ was not released from the cell. To corroborate results, confocal microscopy was employed and revealed that SLPI was localized diffusely and uniformly throughout the cytosol of control resting cells (green). In contrast, after fMLP/IL-8 or PMA stimulation for 10 min the majority of SLPI translocated to the periphery of the cell (Figure 8(c)). Moreover, a greater level of SLPI secretion from CF and COPD neutrophils (5 × 10^6^/mL) was detected in extracellular supernatants by Western blot analysis (Figure 8(d)). This latter result is in agreement with data indicating a requirement for increased SLPI for modulation of COPD and CF neutrophil activity (Figures 2(c) and 2(d)) possibly due to the occurrence of cell priming. On the assumption that extracellular release of SLPI must follow the same time course of seconds as Ca^2+^ flux, we monitored release of SLPI from the cell after 10, 20, 30, 40, and 50 sec fMLP/IL-8 stimulation. Release of SLPI was quantified in the extracellular supernatant by immunoblotting and results demonstrate that levels of extracellular SLPI were significantly increased after just 10 sec stimulation (Figure 8(e), *P* < 0.05). Although the time course of Ca^2+^ flux peaked at 10 sec, a lag period of 30 sec for the maximum level of detectable cell free SLPI was evident. This may in part be explained by the time required for immunodetection of measurable amounts of the protein on the outside of the cell. 

SLPI release from the cell following activation has been comprehensively studied [11]; however the signalling events including possible phosphorylation of SLPI and necessary membrane receptor interactions required for its release, have yet to be evaluated. Nevertheless, collectively these results indicate that upon exposure to proinflammatory stimuli cytosolic SLPI is actively secreted from the cell, and the time of elimination of SLPI coincides with the time of Ca^2+^ flux. 

## 4. Conclusion

**Figure 9:**
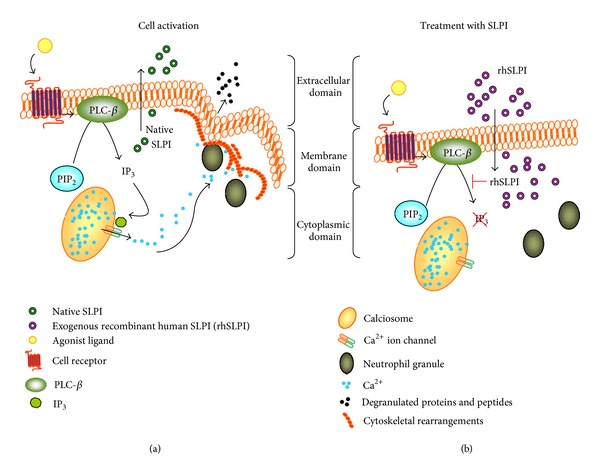
SLPI regulates neutrophil activity by modulating IP_3_ production. (a); Upon neutrophil stimulation SLPI is secreted from the cell to the extracellular environment and IP_3_ is formed during PLC-*β* activation. IP_3_ binds to Ca^2+^ voltage-gated channels on calciosomes (or endoplasmic reticulum) and allows Ca^2+^ ions to flow from the lumen of the calciosome toward the cell interior. The presence of Ca^2+^ ions within the cytosol allows cytoskeletal rearrangements such as actin polymerization and talin cleavage facilitating the process of cell degranulation and chemotaxis. (b); Exogenously added rhSLPI effectively accumulates within the cell and reduces IP_3_ production thereby inhibiting degranulation of antimicrobial peptides and proteins and cell migration.

This study presents evidence that SLPI is present in the cytosol and also the secondary granules of resting neutrophils and is secreted from the cell upon exposure to the phagocyte activator PMA or the chemokines IL-8 and fMLP. We show that neutrophil cytosolic SLPI is effective as a serine protease inhibitor with potent activity against NE. Moreover, within the neutrophil SLPI maintains the ability to modulate cellular processes involving cytoskeletal restructuring including directional chemotaxis and degranulation of antimicrobial peptides and proteases. This investigation elucidates a hitherto undescribed function of intracellular neutrophil cytosolic SLPI and indicates that the described anti-inflammatory effects of SLPI may be orchestrated through inhibition of Ca^2+^ flux by modulating IP_3_ production (Figure 9). Due to the correlative relationship between neutrophil activation and cytosolic Ca^2+^ levels, inhibition of Ca^2+^ flux by SLPI may pose as a potential anti-inflammatory therapy during acute exacerbation of severe chronic obstructive lung disease, thereby reducing neutrophil influx into the airways.

SLPI, elafin, and AAT have been a focus of interest from a therapeutic viewpoint for a number of years. Modifying excessive neutrophil activation and an overexuberant inflammatory response would be a relevant treatment objective in a wide variety of conditions including COPD and CF and also in the modulation of periodontal disease, sepsis syndromes, asthma, bronchiectasis, and in transplant rejection. Recent studies have employed aerosolized liposomal [62] and hydrogel [63] formulations for successful delivery of SLPI. Our finding demonstrating the inhibitory capacity of SLPI on IP_3_ production and Ca^2+^ flux strengthens its attractiveness as a potential therapeutic, and the finding that SLPI has an anti-inflammatory role via inhibition of neutrophil chemotaxis and degranulation is a novel concept that opens up a new field of investigation. 

## Supplementary Material

Supplementary Data: An evaluation of the effect of SLPI compared to calpastatin on calpain activity. The inhibition constant (Ki) of SLPI and calpastatin (endogenous calpain inhibitor) against calpain 1 (Supplementary Figure 1 A and C) and calpain 2 (Supplementary Figure 1 B and D) activity was quantified. GraphPad Prism version 4.03 was employed to calculate Ki values with global curve fit performed finding the single best-fit estimate. Each line represents nonlinear regression analysis of simulated rates of metabolite formation (velocity) as a function of substrate concentration [S]. Results demonstrated the Ki value of calpastatin on calpain 1 and 2 activity as 5.548nM and 1.548nM, respectively (Supplementary Figure 1 A and B). SLPI had no detectable inhibitory effect on calpain 1 and 2 activity resulting in no quantifiable inhibitory constant (Supplementary Figure 1C and E).

## Abbreviations

SLPI: Secretory leukoprotease inhibitor
COPD: Chronic obstructive pulmonary disease
CF: Cystic fibrosis
IP_3_: Inositol 1,4,5-triphosphate
NE: Neutrophil elastase.
