# Supplementary material for: Intracellular Secretory Leukoprotease Inhibitor Modulates Inositol 1,4,5-Triphosphate Generation and Exerts an Anti-Inflammatory Effect on Neutrophils of Individuals with Cystic Fibrosis and Chronic Obstructive Pulmonary Disease
Source: Biomed Res Int. 2013 Aug 29;2013:560141. doi: 10.1155/2013/560141 (PMC3773400; doi:10.1155/2013/560141)
Supplement: Supplementary file 1 — Supplementary Data: An evaluation of the effect of SLPI compared to calpastatin on calpain activity. The inhibition constant (Ki) of SLPI and calpastatin (endogenous calpain inhibitor) against calpain 1 (Supplementary Figure 1 A and C) and calpain 2 (Supplementary Figure 1 B and D) activity was quantified. GraphPad Prism version 4.03 was employed to calculate Ki values with global curve fit performed finding the single best-fit estimate. Each line represents nonlinear regression analysis of simulated rates of metabolite formation (velocity) as a function of substrate concentration [S]. Results demonstrated the Ki value of calpastatin on calpain 1 and 2 activity as 5.548nM and 1.548nM, respectively (Supplementary Figure 1 A and B). SLPI had no detectable inhibitory effect on calpain 1 and 2 activity resulting in no quantifiable inhibitory constant (Supplementary Figure 1C and E). [file 560141.f1.pdf]

Supplementary Figure 1

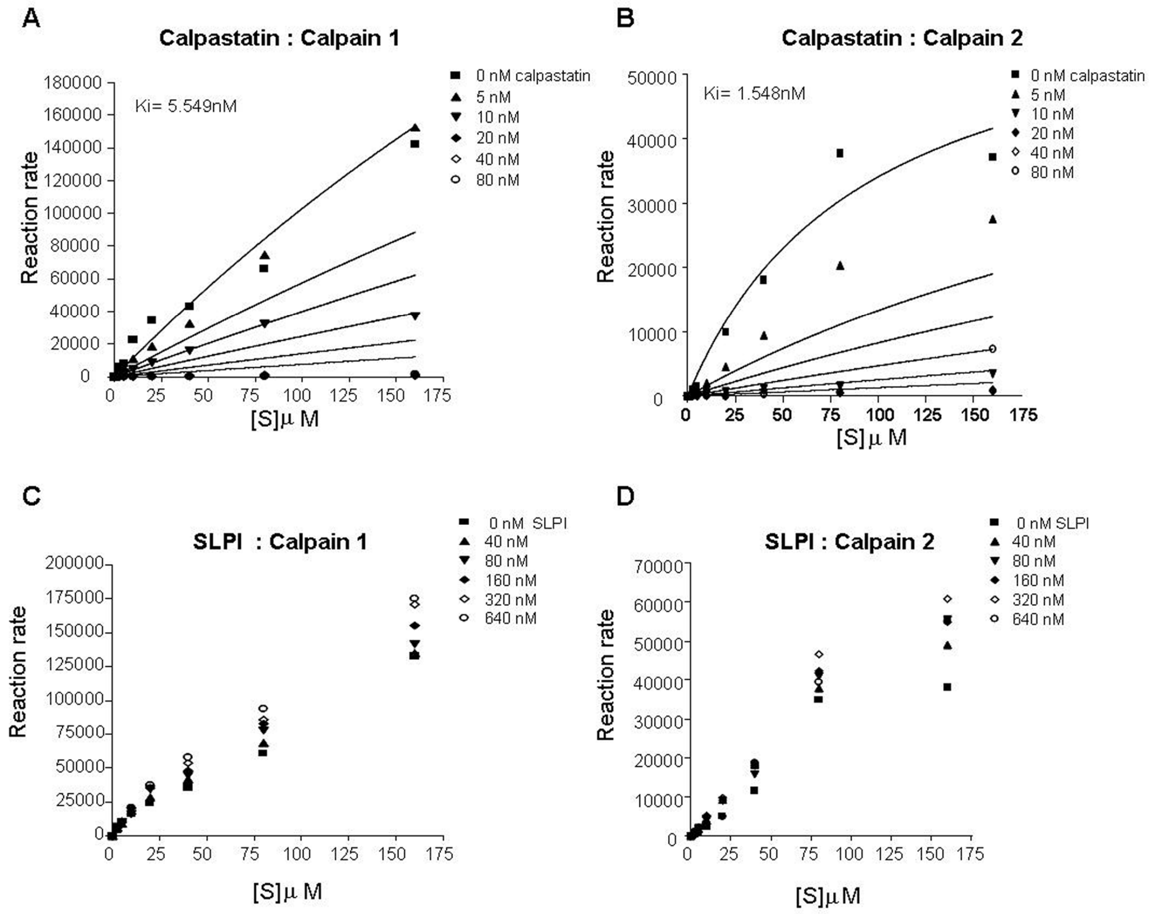

Supplementary Figure 1. SLPI does not inhibit calpain 1 or calpain 2. To determine whether SLPI inhibits calpain 1 or calpain 2 in a competitive manner, the Ki (inhibitor constant) value was determined using the Calpain-Glo(TM) Protease Assay Kit. When compared to the endogenous calpain 1 and 2 inhibitor calpastatin (**A** and **B**), SLPI had no inhibitory effect on calpain 1 or calpain 2 (**C** and **D**).
